# Unraveling the role of 3-mercaptopyruvate sulfurtransferase-derived hydrogen sulfide in triple-negative breast cancer chemoresistance

**DOI:** 10.3389/fphar.2026.1748950

**Published:** 2026-02-09

**Authors:** Sousanna Hakim, Danira A. Habashy, Kelly Ascenção, Rana A. Youness, Carole Bourquin, Csaba Szabo, Mohamed Z. Gad, Reham M. Abdelkader

**Affiliations:** 1 Department of Pharmacology and Toxicology, Faculty of Pharmacy and Biotechnology, German University in Cairo, Cairo, Egypt; 2 Department of Clinical Pharmacy, Faculty of Pharmacy and Biotechnology, German University in Cairo, Cairo, Egypt; 3 Pharmacology Unit, Department of Oncology, Microbiology and Immunology, Section of Science and Medicine, University of Fribourg, Fribourg, Switzerland; 4 Department of Molecular Biology and Biochemistry, Faculty of Biotechnology, German International University, Cairo, Egypt; 5 Department of Biochemistry, Faculty of Pharmacy and Biotechnology, German University in Cairo, Cairo, Egypt; 6 Department of Anesthesiology, School of Pharmaceutical Sciences, Institute of Pharmaceutical Sciences of Western Switzerland, Pharmacology, Intensive Care and Emergency Medicine, University of Geneva, Geneva, Switzerland; 7 Institute of Pharmacology, University of Bern, Bern, Switzerland

**Keywords:** 3-MST, chemoresistance, doxorubicin, H_2_S, TNBC

## Abstract

**Background:**

Triple-negative breast cancer (TNBC) frequently develops resistance to chemotherapy. Cancer-supporting roles of the endogenous gaseous mediator hydrogen sulfide (H_2_S) have been identified. We investigated whether endogenous H_2_S, produced by 3-mercaptopyruvate sulfurtransferase (3-MST), mediates chemoresistance in TNBC and elucidated the underlying mechanisms involved.

**Methods:**

A 3-MST inhibitor (HMPSNE) was used along with different chemotherapeutic drugs to determine whether 3-MST affects TNBC cell (MDA-MB-231) chemoresistance. H_2_S production was measured via AzMC fluorescence. H_2_S-synthesizing and H2S-degrading enzymes were quantified via Western blotting together with downstream signaling molecules involved in the PI3K/Akt/mTOR pathway. Cell viability, colony formation and migration assays were performed. qRT‒PCR and flow cytometry were conducted to assess the expression of the cancer stem cell marker CD44.

**Results:**

HMPSNE enhanced the cytotoxic, anticlonogenic and antimigratory effects of doxorubicin on MDA-MB-231 cells. Doxorubicin increased H_2_S-synthesizing enzymes, whereas HMPSNE resulted in their downregulation, especially cystathionine beta-synthase (CBS) and 3-MST. A similar trend was observed for H_2_S-metabolizing enzymes, particularly thiosulfate sulfurtransferase (TST). A significant increase in CD44 was revealed upon doxorubicin treatment; 3-MST slightly affected this response. With respect to the PI3K/AKT/mTOR pathway, HMPSNE did not significantly modulate the effect of doxorubicin.

**Conclusion:**

These findings suggest that TNBC chemoresistance is linked to the 3-MST/H2S pathway. Pharmacological inhibition of 3-MST by HMPSNE enhances the chemotherapeutic effect of doxorubicin on TNBC. Some of these effects may be related to the regulation of CD44 but are unlikely to be mediated via the PI3K/AKT/mTOR pathway. Therefore, pharmacological inhibition of 3-MST may serve as a promising target for further investigations to increase the sensitivity of TNBC cells to doxorubicin-based therapies.

## 1 Introduction

Triple-negative breast cancer (TNBC) represents a substantially diverse and aggressive subtype of breast cancer (BC), accounting for 10%–20% of all BC cases (Monaco et al., 2023). TNBC is of major clinical interest because of its heterogeneous nature, limited therapeutic modalities, high incidence of metastasis, and poor prognosis (Dent et al., 2007). Standard chemotherapy, which mostly consists of regimens based on anthracyclines and taxanes, is currently the cornerstone of TNBC therapy. (Furlanetto and Loibl, 2020), the lack of estrogen receptor (ER), progesterone receptor (PR) and epidermal growth factor (HER-2) makes TNBC unresponsive to endocrine hormonal therapy or anti-HER-2-based targeted therapy (Al-Mahmood et al., 2018). However, the frequent development of chemoresistance is a fundamental challenge of TNBC therapy (Gooding and Schiemann, 2020; Zeichner et al., 2016). According to the recommendations of the National Comprehensive Cancer Network, doxorubicin and paclitaxel are the most often prescribed chemotherapeutic medications for TNBC (NCCN Clinical Practice Guidelines in Oncology, 2021). However, TNBC patients often develop resistance to these drugs (Mattioli et al., 2023; Xu et al., 2023). Therefore, these drugs were selected for this study together with another anticancer therapy, capecitabine, which was shown to effectively prolong disease-free survival in TNBC patients following neoadjuvant chemotherapy (Shibata and Hoque, 2019).

The mechanisms implicated in TNBC chemoresistance are diverse and include those involving transformation into cancer stem cells (CSCs), which can initiate tumorigenesis, recurrence and metastasis (Shibata and Hoque, 2019; Testa et al., 2018). In TNBC tissues, the proportion of CSCs is greater than that in other subtypes of BC; moreover, in TNBC cells, the CSC population is associated with increased proliferation, migration, invasion and tumorigenicity, indicating its significant role in the aggressive and chemoresistant behavior of TNBC (Ma et al., 2014; Moreira et al., 2018). Oncogenic signaling pathways, such as the JAK/STAT3 and PI3K/AKT/mTOR pathways, are also implicated in TNBC chemoresistance (Nedeljkovic and Damjanovic, 2019). AKT1 and PTEN are mutated in primary TNBC, and these mutations account for 25% of the mutations that occur in primary TNBC (Shah et al., 2012). In addition, AKT1 phosphorylation and, consequently, activation are much more significant in TNBC than in the luminal subtype (Cancer Genome Atlas, 2012). Importantly, high expression of activated AKT was found to be associated with chemoresistance in BC (Steelman et al., 2008). Notably, controlling the AKT signaling pathway restored the sensitivity of TNBC cells to paclitaxel (Han et al., 2018). Moreover, glycogen synthase kinase 3 beta (GSK-3β), a downstream protein of AKT, plays an important role in BC tumorigenicity and chemoresistance, as it promotes resistance to doxorubicin in addition to enhancing clonogenicity and cell signaling in hormone-positive BC cells (McCubrey et al., 2016). GSK-3β could serve as a potential target in TNBC, as GSK-3β inhibitors were found to inhibit CSC properties, suggesting that it is a potential treatment for a subset of aggressive TNBC (Vijay et al., 2019).

**FIGURE 1:**
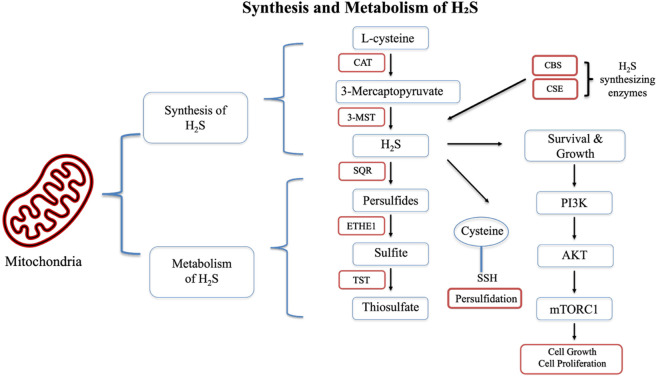
Synthesis and metabolism of H_2_S, with a special emphasis on 3-MST and signaling pathways involved in cell growth and proliferation.

Hydrogen sulfide (H_2_S) is an important mammalian gasotransmitter (Youness et al., 2021; Fahmy et al., 2022; Youness et al., 2024; Dawoud et al., 2024). Recent studies have demonstrated its role in tumorigenesis, tumor cell growth and proliferation (Fahmy et al., 2022; Youness et al., 2018). Three primary enzymes are known to synthesize H_2_S in mammalian tissues: cystathionine-β-synthase (CBS), cystathionine-γ-lyase (CSE) and 3-mercaptopyruvate sulfurtransferase (3-MST) (Szabo, 2007; Kimura, 2011). The levels of H_2_S in a cell or tissue are regulated both by its production and by its catabolism. The latter is mediated by several enzymes, including thiosulfate sulfurtransferase (TST) and persulfide dioxygenase (ETHE1) (Figure 1) (Cirino et al., 2023). The importance of cancer cell-derived H_2_S is evidenced by the upregulation of H_2_S-synthesizing enzymes in numerous cancer cell lines and tissues, including colon, ovarian, melanoma, gastric cancer, and BC (Youness et al., 2021; Szabo et al., 2013; Bhattacharyya et al., 2013; Jurkowska et al., 2011; Zhang et al., 2015). Importantly, heightened sensitivity to chemotherapy has been linked to H_2_S inhibition. HT29 and DLD-1 colon cancer cells treated with AOAA, a pharmacological inhibitor of CBS/CSE, exhibited increased sensitivity to 5-fluorouracil treatment (Chen et al., 2019). AOAA was also found to sensitize colon cancer cells to oxaliplatin (Yue et al., 2020).

H_2_S regulates a number of signaling pathways, including the PI3K/AKT/mTOR pathway (Figure 1). This effect was clearly demonstrated when a CSE inhibitor was shown to inhibit TNBC cell growth through targeting the Ras/Raf/ERK and PI3K/AKT pathways (Liu et al., 2021). Additionally, CD44 is associated with H_2_S signaling: H_2_S donors decrease breast cancer stem cell proliferation through the suppression of CD44 expression (Xie et al., 2018; Zhang et al., 2022). Since the pathways involved in mediating TNBC chemoresistance are apparently involved in H_2_S signaling in TNBC, we decided to examine whether H_2_S plays a role in mediating chemoresistance in TNBC. We focused on one particular H_2_S-producing enzyme, 3-MST. To the best of our knowledge, the available information on the role of this enzyme in TNBC is very limited. According to a recent study by our team, 3-MST is more abundant in BC tissues than in surrounding normal tissues (Dawoud et al., 2024). Moreover, recent work highlighted the role of 3-MST in murine BC and human colon cancer cells (Santos et al., 2023; Augsburger and Szabo, 2020; Ascencao et al., 2022). Thus, the primary goals of the present study were to elucidate whether 3-MST inhibition via 2-[(4-hydroxy-6-methylpyrimidin-2-yl)sulfanyl]-1-(naphthalen-1-yl)ethan-1-one (HMPSNE) can reduce TNBC cell chemoresistance and characterize some of the underlying mechanisms.

## 2 Materials and methods

### 2.1 Cell culture and drug treatment

The human TNBC cell line MDA-MB-231 (obtained from Nawah Scientific, Egypt, which supplies cells from ATCC) was cultured in Dulbecco’s modified Eagle’s medium (DMEM) (Biowest, France) supplemented with 10% fetal bovine serum (FBS) (Biowest, France), 4.5 g/L glucose, 1% penicillin‒streptomycin, and L-glutamine. The cells were kept at 5% CO_2_ and 37 °C (Schmittgen and Livak, 2008). In accordance with the experimental protocol, MDA-MB-231 cells were seeded in 96-, 24-, or 6-well plates and treated with various concentrations of chemotherapeutic agents (doxorubicin, capecitabine, or paclitaxel; MedChemExpress, United States) and/or the 3-MST inhibitor HMPSNE (Molport, Latvia) (Li et al., 2017). Doxorubicin and capecitabine were dissolved in DEPC water, and paclitaxel and HMPSNE were dissolved in DMSO. The concentration of DMSO did not exceed 0.001%. This concentration was tested, and no toxicity was observed.

### 2.2 3-(4,5-Dimethylthiazol-2-yl)-2,5-diphenyl–2H-tetrazolium bromide (MTT) cell viability assay

MDA-MB-231 TNBC cells (20,000 cells/well) were seeded in 96-well plates and treated the next day according to the experimental treatment groups as follows: the control group, drug-only treatment group, drug- and HMPSNE-treated group, and HMPSNE-only treatment group.

The cell culture medium was removed after 48 h. MTT powder (0.1 g) was dissolved in 20 mL of phosphate-buffered saline (PBS) to prepare the MTT stock solution. For each experiment, the stock solution was diluted 1:10 in DMEM supplemented with 10% FBS to obtain the MTT working solution. A volume of 200 µL of the MTT working solution was then added to each well containing MDA-MB-231 cells. The mixture was then incubated for 4 h. Following incubation, the formazan crystals were dissolved in 200 µL of a DMSO/ethanol solution (1:1, v/v) prior to absorbance measurement. Absorbance was measured at 595 nm (Li et al., 2018). The experiments were performed in triplicate and independently repeated three times.

### 2.3 Measurement of H_2_S levels

In black 96-well plates, MDA-MB-231 cells (20,000 cells/well) were seeded and subjected to the previously mentioned treatments (see the MTT cell viability assay Section 2.2). Hydrogen sulfide levels were measured using 7-azido-4-methylcoumarin (AzMC; Sigma-Aldrich, Germany). AzMC was initially dissolved in DMSO and subsequently diluted in HBSS to achieve a final working concentration of 100 μM, with a final DMSO concentration of 0.1%.

A volume of 100 µL of the AzMC working solution was added to the cells, followed by incubation for 1 h at 37 °C.

The fluorescence was measured via a Victor multilabel plate reader at 365 and 450 nm for excitation and emission, respectively (Santos et al., 2023). The experiments were performed in triplicate and independently repeated three times.

### 2.4 Colony-forming assay

In 24-well plates, MDA-MB-231 cells (60,000 cells/well) were seeded, subjected to treatment (see the MTT cell viability Section 2.2), and then incubated for 48 h. To allow colonization, 1,000 cells from each treatment group were trypsinized from the initial cell culture and then, plated per well on a 6-well plate and cultured for 7–10 days. After fixation with 6% glutaraldehyde, the colonies were stained with 0.5% crystal violet. The colonies were manually counted (Youness et al., 2021). The experiments were performed in triplicate and independently repeated three times.

### 2.5 Migration assay

The *in vitro* migration capacity of the cells was assessed via a modified Boyden chamber assay (BD Biosciences, Bedford, MA, United States). MDA-MB-231 cells were initially seeded in 24-well plates at a density of 60,000 cells/well and treated with HMPSNE, doxorubicin, or their combination according to the experimental design. After 48 h of treatment, the cells were trypsinized, counted, and resuspended in low-serum medium (DMEM supplemented with 1% FBS). Transwell inserts with a 5 µm pore size were placed into 24-well plates. A cell suspension containing 50,000 cells in 300 µL of low-serum medium was added to the upper chamber of the Transwell for each treatment group. The lower chamber was filled with 700 µL of high-serum medium (DMEM supplemented with 20% FBS) to serve as a chemoattractant. After 20–24 h of incubation, non-migrated cells were removed from the upper surface of the membrane using a cotton swab. The migrated cells on the lower surface were fixed and stained with 1% crystal violet for 30 min. The membranes were visualized via an inverted brightfield microscope. Following image acquisition, the crystal violet was eluted by transferring the inserts into 700 µL of 2% ethanol. The optical density was quantified by transferring 200 µL of the eluted dye in triplicate to a 96-well plate, and the absorbance was measured at 595 nm via a Victor 1420 multilabel plate reader. The experiments were performed in triplicate and independently repeated three times.

### 2.6 Total RNA extraction and reverse transcription

Total RNA was extracted from MDA-MB-231 cells via the Biozol reagent (Invitrogen, United States). Using a RevertAid First Strand cDNA Synthesis Kit (Thermo Scientific, United States), reverse transcription was carried out according to the manufacturer’s instructions (Bullock et al., 2024).

### 2.7 Quantitative RT‒PCR

The relative quantification method was used to analyze the expression of CD44 via the StepOne™ Plus Real-Time (RT) PCR system (Applied Biosystems, United States). The following TaqMan® real-time PCR assay was used: CD44 (Thermo Fisher Scientific, Hs01075864_m1).

The housekeeping gene used was β-actin, which was labeled with VIC reporter dye (Hs01060665_g1). Relative quantification was performed via the 2^−ΔΔCT^ method (Schmittgen and Livak, 2008).

### 2.8 Flow cytometry

After being seeded in 6-well plates at 150000 cells per well, the MDA-MB-231 cells were treated in the same manner as described in Section 2.2 and incubated for 48 h. Following trypsinization, the cells underwent two rounds of washing before being resuspended in PBS containing 1% FBS (10^6^ cells/100 µL). The cells were then treated with a rat anti-CD44 FITC-conjugated antibody (Sigma Aldrich, United States) for 30 min at 4 °C. After washing, the labeled cells were resuspended in PBS containing 1% FBS. FACS analysis was carried out via Beckman Coulter’s CytoFLEX. CytExpert software was used to analyze the data (Alkaraki et al., 2020).

### 2.9 Western blotting

The cells were scraped from the well plate surface and lysed via RIPA lysis buffer (Thermo Fisher Scientific) supplemented with 1x Halt™ Protease and Phosphatase Inhibitor Cocktail (Thermo Fisher Scientific). The lysates were sonicated for 15 s in an ultrasonic bath (XUBA3, Grant, United Kingdom). Protein concentrations were measured via a BCA protein assay kit (Thermo Fisher Scientific) on a SpectraMax iD5 (Molecular Devices, CA, United States). Proteins were reduced and denatured by boiling in lithium dodecyl sulfate (LDS) sample buffer (Bolt™ LDS Sample Buffer, Invitrogen, Thermo Fisher Scientific) or (NuPAGE™ LDS Sample Buffer, Invitrogen) at 95 °C for 5 min. Equivalent amounts of protein were then loaded and separated on Bolt™ 4%–12% Bis-Tris Plus Gels (Invitrogen) or NuPAGE™ 3%–8% Tris-acetate Gels under a constant voltage of 100 V. NuPAGE™ 3%–8% Tris-Acetate gels were used only for detecting the mTORC signals as this gel system is specifically designed for high-molecular-weight proteins and allowed efficient separation and detection of mTOR. However, all other proteins were detected using Bolt™ 4%–12% Bis-Tris gels as it is the preferred for broad-range and smaller proteins.

Proteins were transferred to PVDF membranes via dry transfer via the iBlot™ 2 System (Invitrogen). The membranes were blocked with 5% milk in TBS/0.1% Tween (TBST) for 1 h at RT. Primary antibodies diluted in 5% BSA-TBST or 5% milk-TBST were incubated for 1 h at RT or overnight at 4 °C. Following incubation, the membranes were washed three times with TBST and then incubated for 1 h at RT with either anti-rabbit IgG or anti-mouse IgG HRP-conjugated secondary antibodies (1:5,000 dilution in 5% milk-TBST). After three additional washes with TBST, the blots were developed via Radiance Plus chemiluminescence (AC2103, Azure Biosystems, CA, United States) on an Azure Imaging System 300 (Azure Biosystems). Signal intensities were quantified via ImageJ software (NIH, Bethesda, MD, United States), with band intensities normalized to those of β-actin as the loading control. Representative blots from at least four independent experiments are shown.

We used the following antibodies: rabbit monoclonal anti-CBS (D8F2P), anti-AKT (pan) (C67E7), anti-p-Akt (Ser473) (D9E), anti-GSK-3β (D5C5Z), mTOR (7C10) and anti-mouse IgG HRP-linked antibodies, which were purchased from Cell Signaling Technology (Danvers, MA, United States). Mouse monoclonal anti-β-actin (AC-15) was obtained from Sigma‒Aldrich (Burlington, MA, United States). Anti-rabbit IgG (H + L) cross-adsorbed secondary antibody-HRP was purchased from Invitrogen (Thermo Fisher Scientific). Rabbit polyclonal anti-3-MST (ab224043), anti-CSE (ab151769), anti-TST (ab231248) and rabbit monoclonal anti-ETHE1 (ab174302) were acquired from Abcam (Cambridge, England).

### 2.10 Statistical analysis

The normality test was performed via the Shapiro‒Wilk test. When two independent groups were compared, an unpaired Student’s t-test was employed for all normally distributed data. For data from more than two groups, ordinary one-way ANOVA was used for all normally distributed data. The means ± standard errors of the means (SEMs) are used to express the data. Statistical significance was defined as * = p < 0.05 and ** = p < 0.01. GraphPad Prism 8.0 software was used for the statistical analysis.

## 3 Results

### 3.1 Cytotoxic effects of chemotherapeutic agents on MDA-MB-231 TNBC cells

**FIGURE 2:**
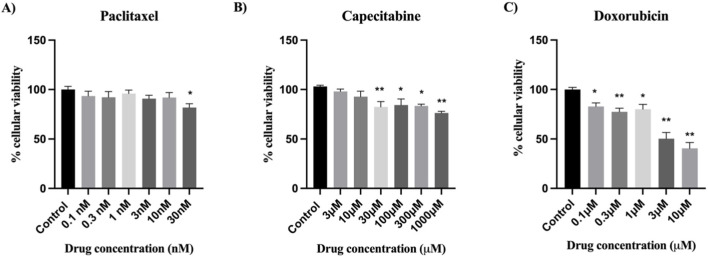
Effects of various concentrations of **(A)** paclitaxel, **(B)** capecitabine and **(C)** doxorubicin on the viability of MDA-MB-231 cells 48 h post-treatment. Cell viability was evaluated via the MTT assay. The data are presented as the means ± SEMs of six independent experiments; * = P < 0.05 and ** = P < 0.01 indicate significant cytotoxic effects.

MDA-MB-231 cells were exposed to different concentrations of doxorubicin, capecitabine, and paclitaxel for 48 h (Augsburger et al., 2020; Ascencao et al., 2021; Hellmich et al., 2015; Kajsik et al., 2022; Stokes et al., 2018; Szabo et al., 2014; Vogel and Marcotte, 2012; Zhang et al., 2024). The percentage of viable cells was calculated relative to the control. MDA-MB-231 cells were resistant to paclitaxel; only at 30 nM did this drug result in a slight decrease in cell viability (Figure 2A). Capecitabine was found to reduce the viability of the cells, and this reduction was statistically significant at concentrations ranging from 30 to 1000 µM (Figure 2B). Compared with other chemotherapeutic agents, doxorubicin decreased cell viability in a concentration range of 0.1–10 µM in a concentration-dependent manner (Figure 2C).

### 3.2 Effect of HMPSNE treatment on the susceptibility of MDA-MB-231 cells to the cytotoxic effects of chemotherapy

**FIGURE 3:**
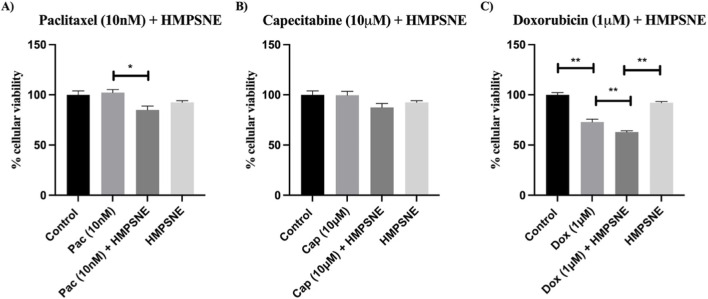
Effects of HMPSNE (100 µM) on the cytotoxicity of **(A)** paclitaxel, **(B)** capecitabine and **(C)** doxorubicin to MDA-MB-231 cells 48 h after treatment. The data are presented as the means ± SEMs of six independent experiments; *P < 0.05 and **P < 0.01 indicate significant differences between the indicated groups.

Next, we tested the effects of inhibiting 3-MST via HMPSNE (100 µM) on the cytotoxic effects of the three chemotherapeutic agents used in our study. The addition of HMPSNE to paclitaxel (10 nM) significantly decreased the percentage of viable cells. (Figure 3A), compared with paclitaxel alone. The addition of HMPSNE (100 µM) to 10 µM capecitabine did not have a significant effect (Figure 3B). The addition of HMPSNE (100 µM) increased the cytotoxic effect of 1 µM doxorubicin (Figure 3C). However, the HMPSNE alone tended to decrease in viability.

### 3.3 Effects of HMPSNE and chemotherapy on H_2_S levels in MDA-MB-231 TNBC cells

**FIGURE 4:**
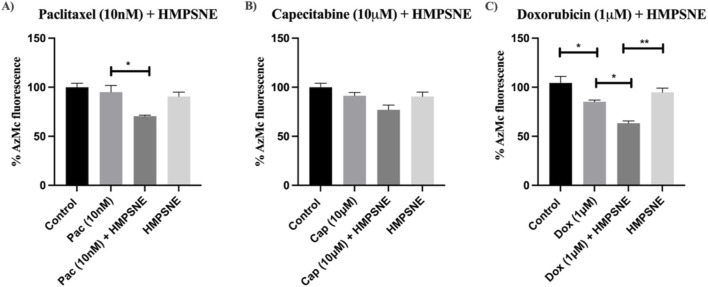
Effects of HMPSNE (100 µM), either alone or in combination with various chemotherapeutic drugs, such as **(A)** paclitaxel, **(B)** capecitabine, **(C)** doxorubicin, on H_2_S levels in MDA-MB-231 cells. The data are presented as the means ± SEMs of three independent experiments; *P < 0.05 and **P < 0.01 indicate significant differences between the indicated groups.

HMPSNE (100 µM) only slightly but nonsignificantly affected H_2_S levels in MDA-MB-231 TNBC cells. Compared with the addition of paclitaxel alone, the addition of HMPSNE (100 µM) to 10 nM paclitaxel (Figure 4A) caused a significant decrease in H_2_S levels. This effect was not observed with capecitabine cotreatment (Figure 4B). In contrast, compared with doxorubicin or HMPSNE single treatments, dual treatment with HMPSNE and doxorubicin significantly reduced H_2_S levels. Notably, doxorubicin alone also decreased H_2_S levels and reached statistical significance relative to those in the control untreated group (Figure 4C).

### 3.4 Effects of HMPSNE and doxorubicin on the protein levels of enzymes that synthesize and metabolize H2S in MDA-MB-231 cells

**FIGURE 5:**
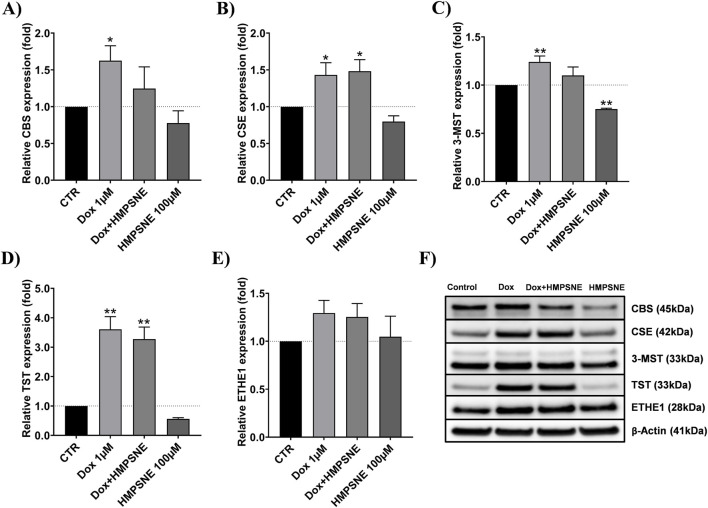
Effects of HMPSNE and/or doxorubicin on the expression of H_2_S-synthesizing and H_2_S-metabolizing enzymes. **(A–E)** show the quantification of the Western blot densitometry; **(F)** shows representative Western blots. The data are shown as the means ± SEMs of four independent experiments: * = P < 0.05, ** = P < 0.01 compared with the untreated control.

However, doxorubicin treatment significantly increased the protein expression of the H_2_S-producing enzymes CBS, CSE, and 3-MST (by approximately 1.5-fold, 1.5-fold, and 1.3-fold, respectively) (Figures 5A–C, F), this was not accompanied by an increase in total H_2_S production. Notably, doxorubicin also markedly upregulated the H_2_S-catabolizing enzyme TST and, to a lesser extent, ETHE1 (by approximately 3-fold and 1.3-fold, respectively) (Figures 5D–F), which may contribute to enhanced mitochondrial H_2_S clearance.

In contrast, HMPSNE treatment reduced 3-MST protein expression by approximately 1.3-fold decrease, indicating partial suppression of the mitochondrial H_2_S-producing pathway (Figure 5C).

### 3.5 Impact of HMPSNE and doxorubicin cotreatment on the colony-forming ability and migration of MDA-MB-231 cells

**FIGURE 6:**
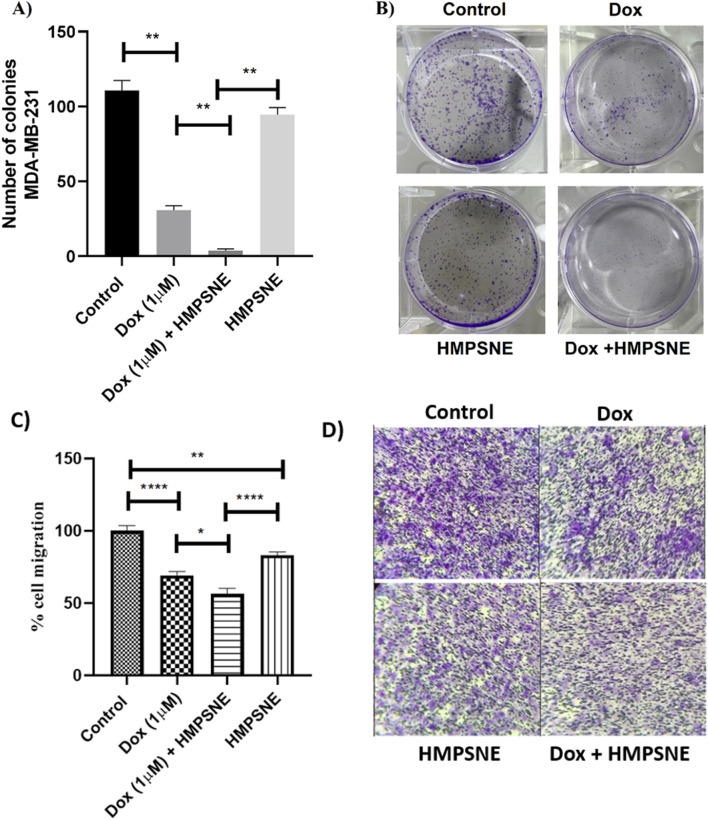
Effects of HMPSNE (100 µM) alone or in combination with doxorubicin (1 µM) on the clonogenicity and migration of MDA-MB-231 cells. **(A,C)** Compared with the doxorubicin- or HMPSNE-treated groups, the combination group presented a significant reduction in the number of colonies formed and in the degree of cellular migration. Compared with the control, doxorubicin significantly reduced the number of formed colonies and the degree of cellular migration. **(B,D)** Representative images of the plates and transwells. The data are presented as the means ± SEMs of three independent experiments: *P < 0.05 and **P < 0.01 indicate statistically significant differences between the indicated groups.

Compared with treatment with either doxorubicin or HMPSNE alone, combined treatment of MDA-MB-231 cells with HMPSNE or doxorubicin led to an almost complete decrease in the number of formed colonies (Figures 6A,B) and a significant decrease in cellular migration (Figures 6C,D). However, compared with control cells, HMPSNE-alone-treated cells tended to have decreased clonogenicity (Figure 6A) and significantly decreased migration (Figure 6C).

### 3.6 Effects of HMPSNE and doxorubicin on CD44 expression in MDA-MB-231 TNBC cells

**FIGURE 7:**
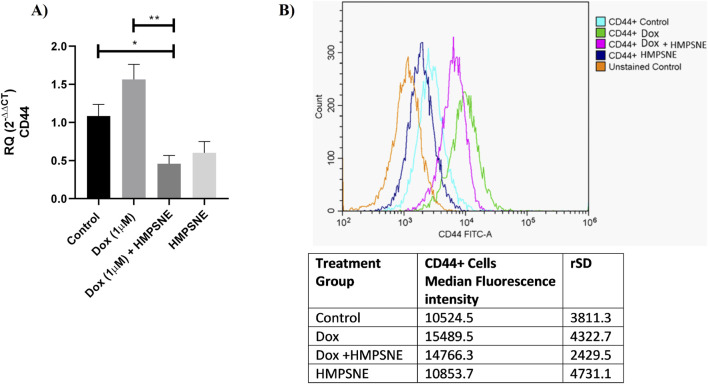
Effects of HMPSNE (100 µM) alone or in combination with doxorubicin (1 µM) on CD44 expression in MDA-MB-231 cells. **(A)** qRT‒PCR was performed, and the results were normalized to those of B-actin as an internal control. The data are shown as the means ± SEMs of three independent experiments: *P < 0.05, **P < 0.01. **(B)** Representative curves for flow cytometry analysis of TNBC cells before and after treatment. The overlay shows CD44 expression upon treatment with doxorubicin and HMPSNE in comparison with the unstained control (orange), which was used to establish baseline autofluorescence and gating thresholds. The median fluorescence values with relative standard deviations are shown in the table under the figure. This experiment was repeated three times.

CSCs, which contribute to the stemness of tumor cells and consequently the resistance of TNBC cells, have been shown to be affected by H_2_S signaling (Li et al., 2017; Li et al., 2018). Therefore, we investigated whether doxorubicin, on its own or in combination with 3-MST inhibition, affects the formation of the CSC surface marker CD44. Figure 7A shows the gene expression profile of CD44 after the treatment of MDA-MB-231 cells with HMPSNE and doxorubicin (1 µM). Compared with doxorubicin treatment alone, the combination of HMPSNE (100 µM) and doxorubicin (1 µM) markedly decreased the quantity of CD44 mRNA transcripts. HMPSNE (100 µM), on its own, was also associated with low expression of CD44 mRNA transcripts. When the protein expression of CD44 was investigated via flow cytometry analysis, doxorubicin treatment clearly increased CD44 expression. CD44 expression in HMPSNE-treated cells was slightly reduced. Compared with treatment with doxorubicin alone, the combination of doxorubicin and HMPSNE caused a slight decrease in CD44 protein expression. CD44 expression in HMPSNE-treated cells was low. However, compared with doxorubicin alone, the combination of doxorubicin and HMPSNE caused only a slight decrease in CD44 protein expression (Figure 7B).

### 3.7 Effects of HMPSNE and doxorubicin on AKT/GSK-3β/mTOR levels

**FIGURE 8:**
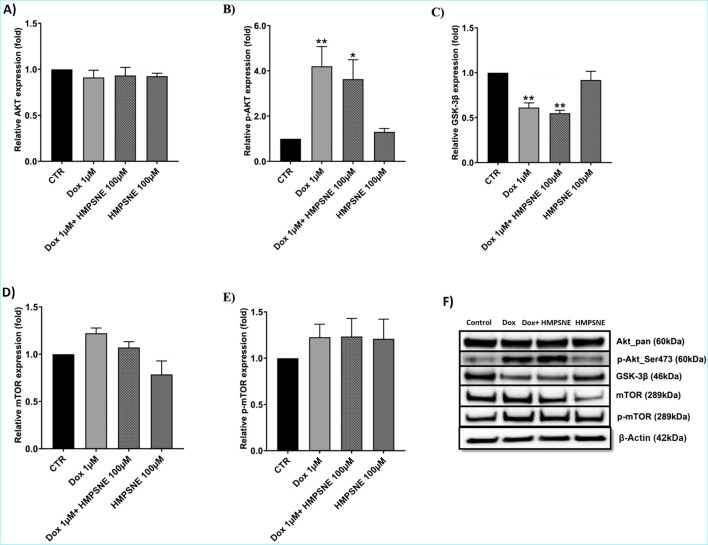
Effects of HMPSNE (100 µM) alone or in combination with doxorubicin (1 µM) on AKT, GSK-3β and mTOR expression in MDA-MB-231 cells. **(A–E)** show the quantification of the Western blot densitometry; **(F)** shows representative Western blots. The data are shown as the means ± SEMs of four independent experiments: * = P < 0.05, ** = P < 0.01 compared with the untreated control.

One of the pathways most commonly dysregulated in TNBC is the PI3K/AKT/mTOR pathway. Mutations or alterations in this pathway occur in almost 25% of TNBC patients (Bullock et al., 2024; Zhang et al., 2024), promoting chemoresistance, in addition to being implicated in H_2_S signaling (Steelman et al., 2008; Liu et al., 2021). Therefore, we quantified the levels of AKT, GSK-3β and mTOR in TNBC cells treated with HMPSNE or doxorubicin. Figures 8A,B,F show the effects of HMPSNE or doxorubicin alone or in combination on the expression levels of AKT and p-AKT. Doxorubicin significantly increased p-AKT levels (Figures 8B,F). The addition of HMPSNE (100 µM) to doxorubicin slightly reduced p-AKT expression (Figures 8B,F). Total AKT protein levels were not altered in any of the treated groups (Figures 8A,F), suggesting an increase in the phosphorylation of existing AKT protein without a change in total AKT protein levels. GSK-3β protein levels were significantly suppressed upon doxorubicin treatment (Figures 8C,F), which may be related to increased p-AKT levels via the inhibition of GSK-3β, which in turn tends to increase mTOR levels, as shown in Figures 8D–F. The inhibition of 3-MST with HMPSNE did not significantly affect the doxorubicin-induced changes in pAKT or GSK-3β expression (GSK-3β) (Figures 8B,C,F). Although combined treatment with doxorubicin and HMPSNE significantly decreased the level of GSK-3β, similar to that of doxorubicin (Figures 8C,F), the level of mTOR tended to decrease compared with that of doxorubicin alone (Figures 8D,F). These observations can be explained by the slight decrease in p-AKT levels (compared with those in doxorubicin alone), which may subsequently reduce mTOR levels.

## 4 Discussion

TNBC is one of the most severe subtypes of BC (Dent et al., 2007), with the only available treatment modality being chemotherapy with anthracyclines and taxanes (NCCN Clinical Practice Guidelines in Oncology, 2021), which has limited efficacy, mainly due to the development of chemoresistance (Mattioli et al., 2023; Xu et al., 2023). The mechanisms of chemoresistance in TNBC are diverse and include those involving the PI3K/AKT/mTOR pathway and cancer stem cells (Shibata and Hoque, 2019; Nedeljkovic and Damjanovic, 2019).

The goal of the current study was to test whether modulation of the endogenous H_2_S system via inhibition of 3-MST affects TNBC viability and responsiveness to chemotherapeutic agents. The reason for selecting this specific H2S-producing enzyme is because our group recently reported that 3-MST is significantly upregulated in breast cancer tissues compared to their normal counterparts (Dawoud et al., 2024). Furthermore, previous studies have demonstrated that 3-MST plays a critical role in supporting tumor growth in murine breast cancer models (Santos et al., 2023; Augsburger and Szabo, 2020). The effects of chemotherapeutic agents (paclitaxel, capecitabine and doxorubicin) on MDA-MB-231 TNBC cells were investigated first, and on the basis of these responses, the effects of doxorubicin were further investigated in detail. The IC_50_ for doxorubicin in MDA-MB-231 cells was in agreement with previously reported values for this cell type (Alkaraki et al., 2020). To determine the effect of H_2_S biosynthesis inhibition on chemotherapeutic sensitivity in TNBC cells, HMPSNE, a specific inhibitor of 3-MST that has been previously used in various cancer cells at a concentration of 100 µM (Augsburger et al., 2020; Ascencao et al., 2021), was used.

Our results showed that the addition of 100 µM HMPSNE to chemotherapy increased the chemotherapeutic effect by decreasing the viability, colony formation ability and migration ability of MDA-MB-231 cells and reducing H_2_S levels in these cells but only in combination with doxorubicin. The fact that HMPSNE, on its own, only exerted a minor effect on the cellular H_2_S level indicates that H_2_S generation in these cells is likely due to a combination of enzymatic and possible nonenzymatic processes and that 3-MST is only one of many contributors. Nevertheless, 3-MST appears to play a significant functional role in this cell type, as evidenced by the colony formation assay and cell viability results presented in this report.

To the best of our knowledge, whether pharmacological inhibition of endogenous H_2_S through 3-MST could enhance the cytotoxicity of doxorubicin in TNBC cells has not been previously investigated. In contrast, previous studies on other cancer models, such as hepatoma (Stokes et al., 2018) and colon cancer cells (Kajsik et al., 2022), described the effect of adding exogenous H_2_S donors, which enhances the efficiency of the chemotherapeutic drugs doxorubicin and paclitaxel, respectively. These latter findings could be attributed to the bell-shaped feature of H_2_S, where high levels of exogenous H_2_S tend to have cytotoxic or antiproliferative effects on cancer cells. However, substantial antiproliferative effects are also observed when the endogenous production of H_2_S is inhibited. For instance, the inhibition of H2S-producing enzymes (CBS or CSE) has been shown to suppress cell proliferation and mitochondrial bioenergetics in colon cancer and ovarian cancer models (Bhattacharyya et al., 2013; Chen et al., 2019). In these contexts, the inhibitors effectively “take away” the endogenous, cell proliferation-supporting roles of H_2_S (Szabo et al., 2014; Hellmich et al., 2015). Unexpectedly, our findings revealed that doxorubicin significantly upregulated the protein levels of CBS, CSE, and 3-MST. This observation aligns with previous reports in HCT116 colon cancer cells, where acquired resistance to 5-fluorouracil was associated with elevated expression of CBS and 3-MST (Zhang et al., 2024). However, despite the upregulation of H2S-synthesizing enzymes, doxorubicin treatment did not result in a net increase in H2S production. This discrepancy is likely attributable to the concurrent, significant upregulation of TST, along with an increasing trend in ETHE1 expression. As TST and ETHE1 are key enzymes responsible for H2S metabolism, their compensatory induction may counterbalance increased H2S synthesis, thereby preventing the accumulation of H2S.

We also noted a decrease in 3-MST expression after HMPSNE treatment, which was not expected because this compound is an inhibitor of activity and not the transcription or translation of this enzyme. Nevertheless, an inhibitory effect of HMPSNE on CBS and 3-MST expression has previously been reported in the HCT116 human cancer cell line, possibly due to the self-amplifying transcriptional role of 3-MST-derived H_2_S (Ascencao et al., 2021).

To understand the mechanism underlying the enhancement of doxorubicin sensitivity, the activation of the PI3K/AKT/mTOR pathway and the expression of CD44, a CSC marker, were studied. These pathways are implicated in TNBC chemoresistance and have been previously linked to the H_2_S pathway in various experimental systems (Moreira et al., 2018; Nedeljkovic and Damjanovic, 2019; Xie et al., 2018; Zhang et al., 2022; Bullock et al., 2024; Zhang et al., 2024). The CD44 expression profile was investigated upon dual treatment with HMPSNE and doxorubicin. Compared with doxorubicin alone, the combination significantly reduced CD44 transcript levels. This finding, however, was not confirmed by flow cytometry analysis of the CD44 protein; at the protein level, doxorubicin increased CD44, and this increase was only slightly reduced by the addition of HMPSNE. The discrepancy between these two findings remains to be explored in the future. This can be mechanistically due to post-transcriptional regulation, translation efficiency, and protein stability (Vogel and Marcotte, 2012). One of the possible explanations specifically mentioned for CD44 in the literature is that the protein undergoes extensive post-translational modifications, including N- and O-glycosylation and palmitoylation, which enhance its stability (Lesle et al., 1993). Consequently, while the combination treatment effectively suppressed CD44 transcription, the high stability of the already present CD44 protein likely resulted in the decreased effect observed in the flow cytometry protein analysis (Pedrycz-Wieczorska et al., 2025). Interestingly, a previous study revealed that exposure of colon cancer cells to exogenous H_2_S suppressed their proliferation and migration through the downregulation of CD44 (Zhang et al., 2022). 3-MST may mediate doxorubicin resistance by expanding the CSC subpopulation and/or upregulating CD44. With respect to the PI3K/AKT/mTOR pathway, HMPSNE treatment did not significantly modulate the effect of doxorubicin; therefore, we conclude that the 3-MST/H_2_S pathway is unlikely to mediate its effects through the modulation of this system under our experimental conditions.

We acknowledge several limitations in the current study that warrant further investigation. First, our findings rely on a single TNBC cell line, MDA-MB-231. While this cell line is a widely accepted gold standard for aggressive, mesenchymal-type TNBC, verifying these results in additional TNBC models and validating the efficacy of the HMPSNE-doxorubicin combination in *in-vivo* TNBC xenograft models will be essential to ensure broader generalizability and relevance. Finally, regarding the specificity of target inhibition, we prioritized a pharmacological approach (HMPSNE) over genetic silencing (siRNA). While genetic knockdown offers high specificity, our primary objective was to better simulate a clinical adjuvant therapy where a pharmacological inhibitor is co-administered with chemotherapy; however, future studies utilizing genetic tools will be valuable to further confirm target specificity.

## 5 Conclusion

In conclusion, the pharmacological inhibition of 3-MST by HMPSNE enhances the chemotherapeutic effect of doxorubicin on TNBC *in vitro*. Some of these effects may be related to the regulation of CD44 but are unlikely to be mediated via the PI3K/AKT/mTOR pathway. Although the exact mechanisms by which HMPSNE enhances doxorubicin responses remain to be further elucidated, the pharmacological inhibition of 3-MST may serve as a promising target for further investigations to increase the sensitivity of TNBC cells to doxorubicin-based therapies.

## Data Availability

The original contributions presented in the study are included in the article/supplementary material, further inquiries can be directed to the corresponding author.
